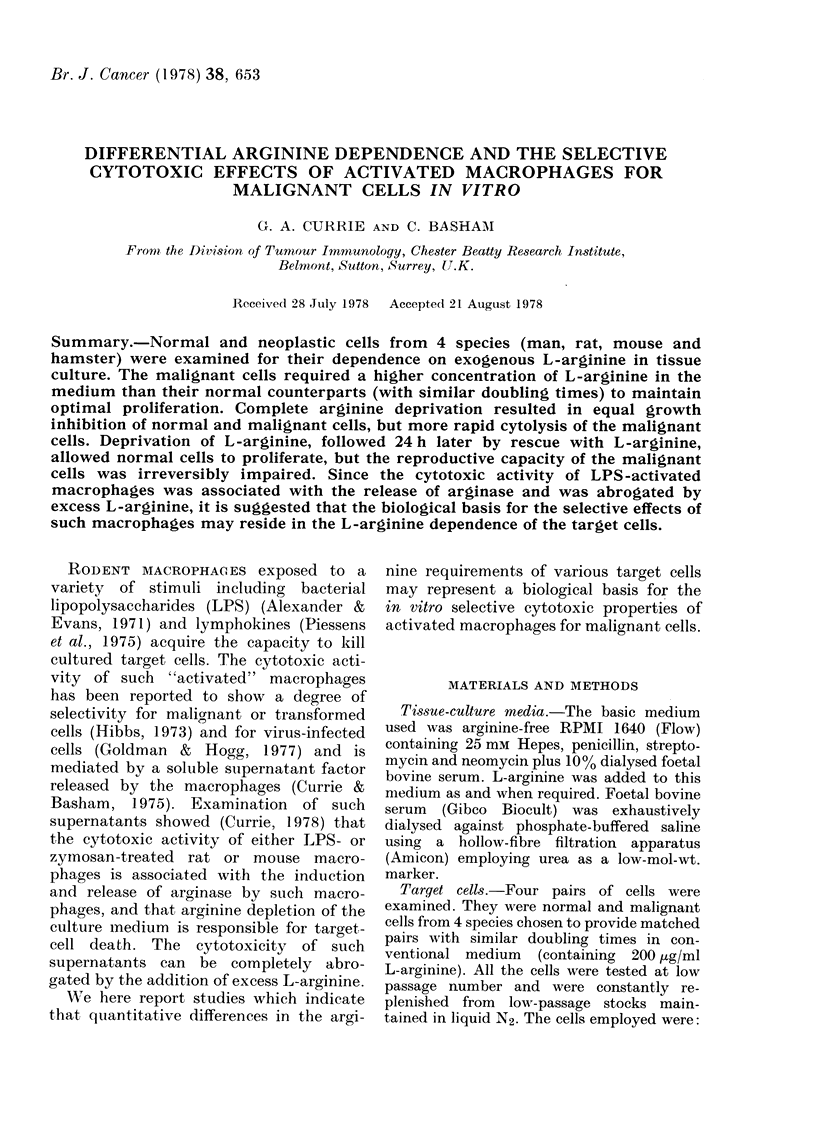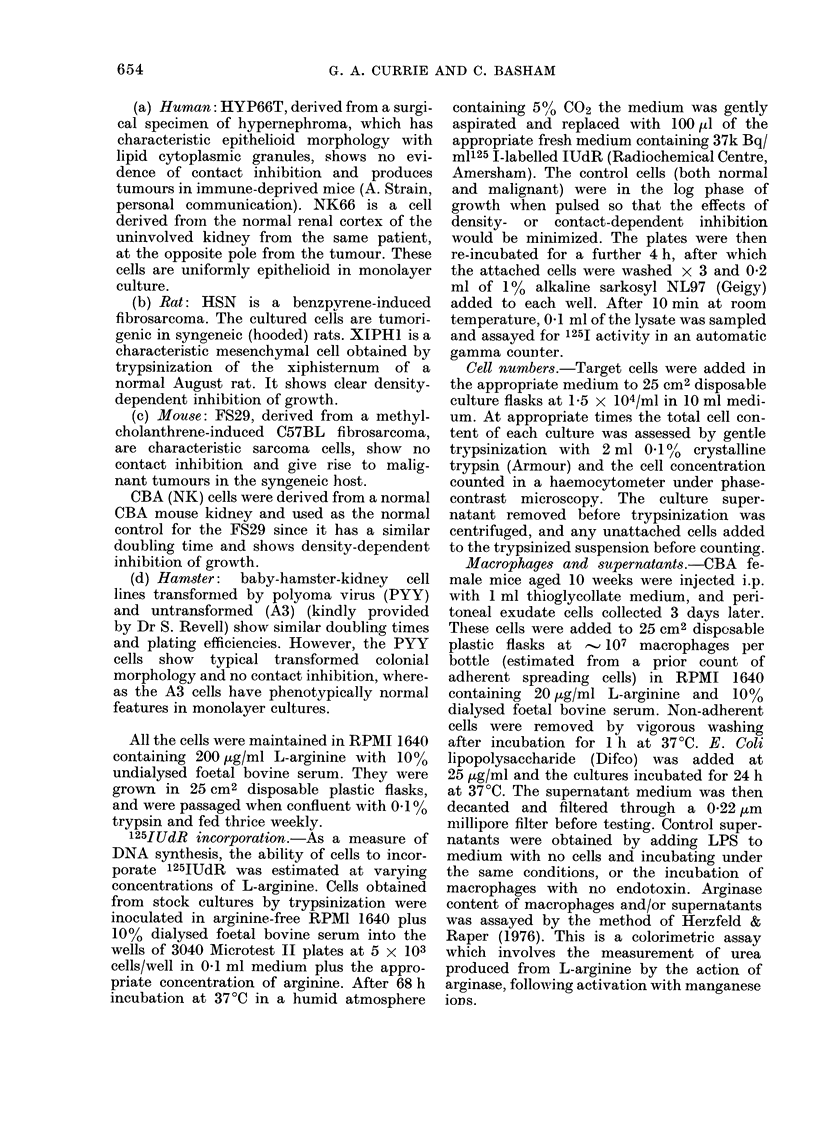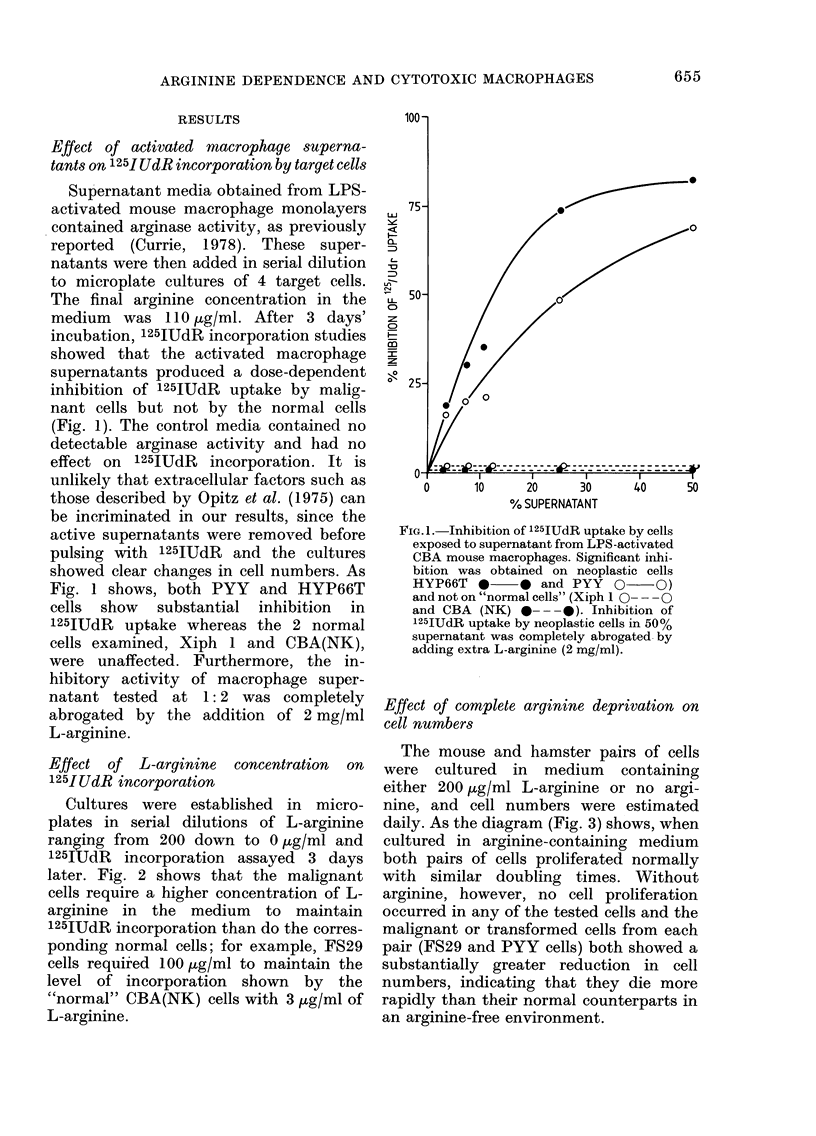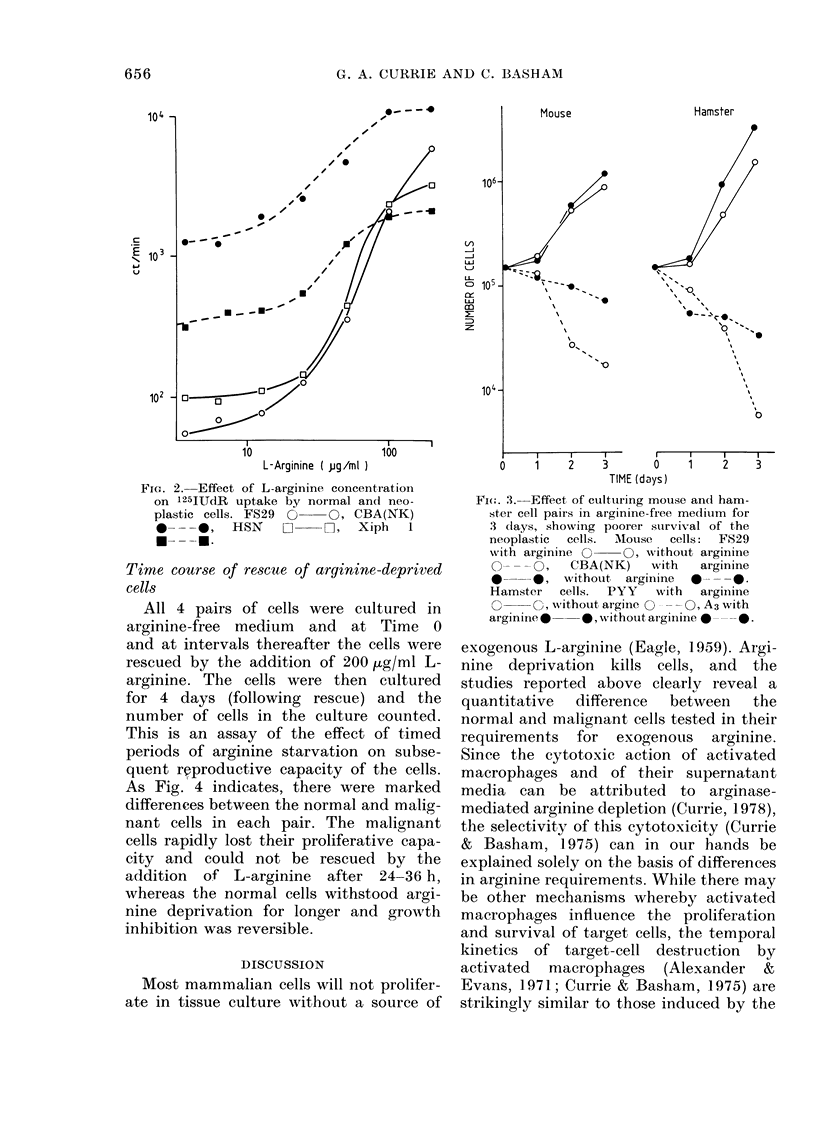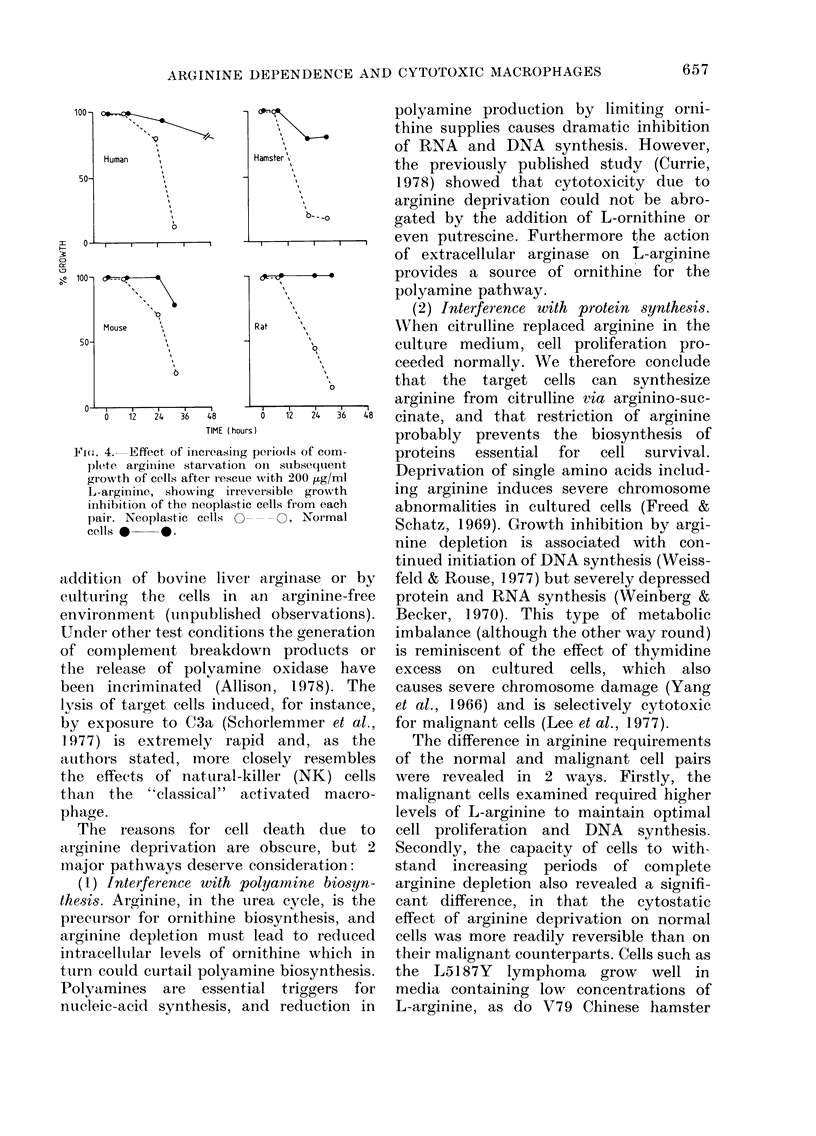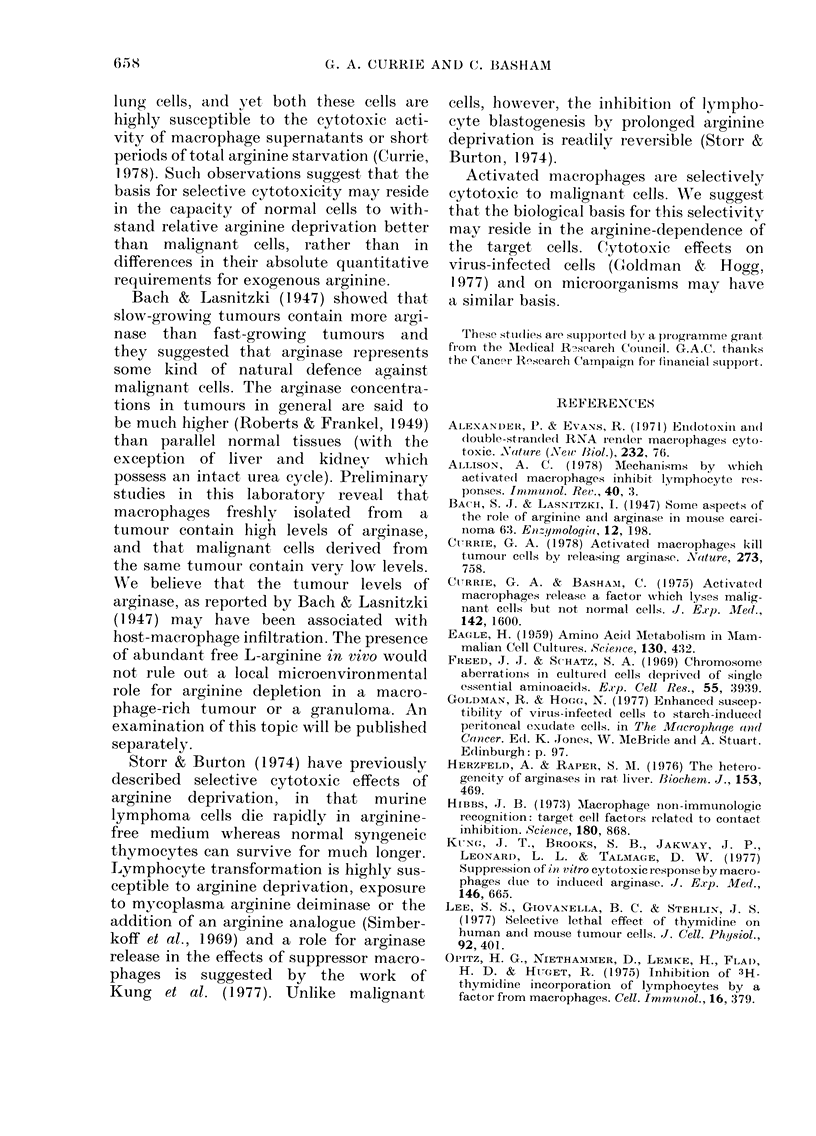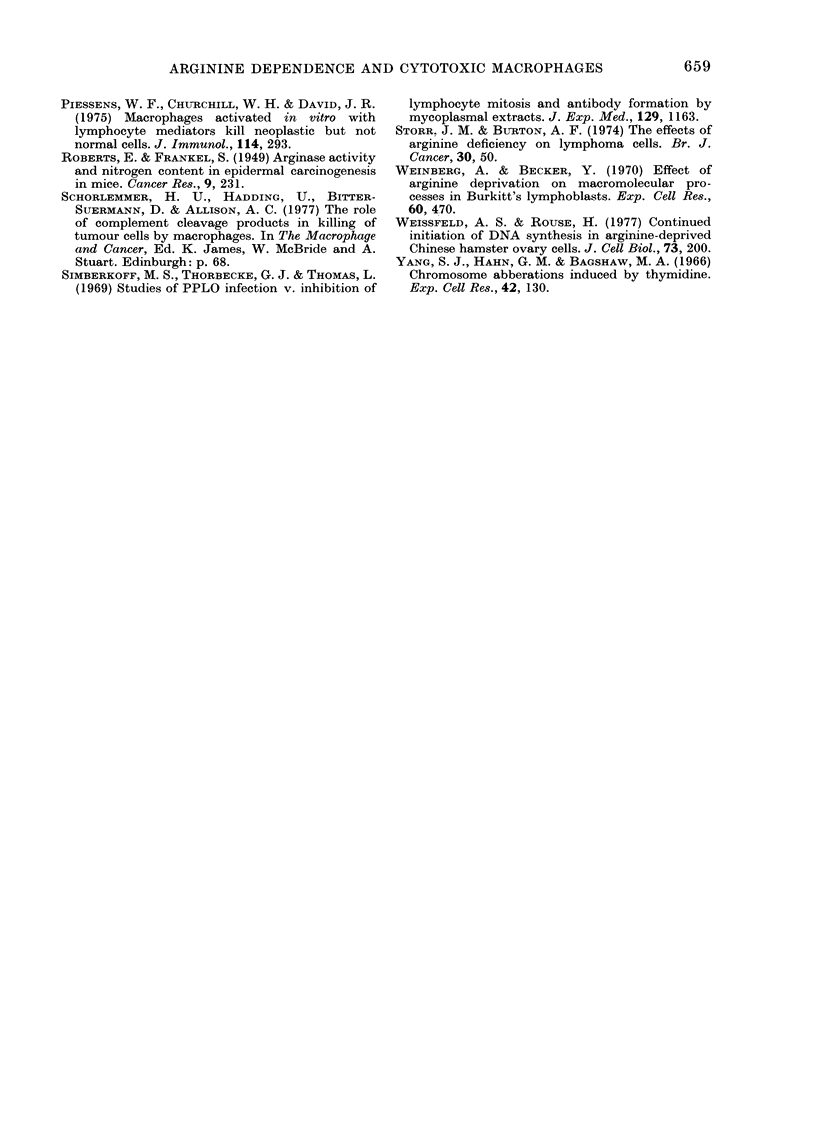# Differential arginine dependence and the selective cytotoxic effects of activated macrophages for malignant cells in vitro.

**DOI:** 10.1038/bjc.1978.270

**Published:** 1978-12

**Authors:** G. A. Currie, C. Basham

## Abstract

Normal and neoplastic cells from 4 species (man, rat, mouse and hamster) were examined for their dependence on exogenous L-arginine in tissue culture. The malignant cells required a higher concentration of L-arginine in the medium than their normal counterparts (with similar doubling times) to maintain optimal proliferation. Complete arginine deprivation resulted in equal growth inhibition of normal and malignant cells, but more rapid cytolysis of the malignant cell. Deprivation of L-arginine, followed 24 h later by rescue with L-arginine, allowed normal cells to proliferate, but the reproductive capacity of the malignant cells was irreversibly impaired. Since the cytotoxic activity of LPS-activated macrophages was associated with the release of arginase and was abrogated by excess L-arginine, it is suggested that the biological basis for the selective effects of such macrophages may reside in the L-arginine dependence of the target cells.


*Br. J. Cancer* (1978) **38**, 653

### DIFFERENTIAL ARGININE DEPENDENCE AND THE SELECTIVE CYTOTOXIC EFFECTS OF ACTIVATED MACROPHAGES FOR MALIGNANT CELLS *IN VITRO*

G. A. CURRIE AND C. BASHAM

*From the Division of Tumour Immunology, Chester Beatty Research Institute, Belmont, Sutton, Surrey, U.K.*

Received 28 July 1978 Accepted 21 August 1978

**Summary.—Normal and neoplastic cells from 4 species (man, rat, mouse and hamster) were examined for their dependence on exogenous L-arginine in tissue culture. The malignant cells required a higher concentration of L-arginine in the medium than their normal counterparts (with similar doubling times) to maintain optimal proliferation. Complete arginine deprivation resulted in equal growth inhibition of normal and malignant cells, but more rapid cytolysis of the malignant cells. Deprivation of L-arginine, followed 24 h later by rescue with L-arginine, allowed normal cells to proliferate, but the reproductive capacity of the malignant cells was irreversibly impaired. Since the cytotoxic activity of LPS-activated macrophages was associated with the release of arginase and was abrogated by excess L-arginine, it is suggested that the biological basis for the selective effects of such macrophages may reside in the L-arginine dependence of the target cells.**

RODENT MACROPHAGES exposed to a variety of stimuli including bacterial lipopolysaccharides (LPS) (Alexander & Evans, 1971) and lymphokines (Piessens *et al.*, 1975) acquire the capacity to kill cultured target cells. The cytotoxic activity of such "activated" macrophages has been reported to show a degree of selectivity for malignant or transformed cells (Hibbs, 1973) and for virus-infected cells (Goldman & Hogg, 1977) and is mediated by a soluble supernatant factor released by the macrophages (Currie & Basham, 1975). Examination of such supernatants showed (Currie, 1978) that the cytotoxic activity of either LPS- or zymosan-treated rat or mouse macrophages is associated with the induction and release of arginase by such macrophages, and that arginine depletion of the culture medium is responsible for target-cell death. The cytotoxicity of such supernatants can be completely abrogated by the addition of excess L-arginine.

We here report studies which indicate that quantitative differences in the arginine requirements of various target cells may represent a biological basis for the *in vitro* selective cytotoxic properties of activated macrophages for malignant cells.

### MATERIALS AND METHODS

*Tissue-culture media.*—The basic medium used was arginine-free RPMI 1640 (Flow) containing 25 mM Hepes, penicillin, streptomycin and neomycin plus 10% dialysed foetal bovine serum. L-arginine was added to this medium as and when required. Foetal bovine serum (Gibco Biocult) was exhaustively dialysed against phosphate-buffered saline using a hollow-fibre filtration apparatus (Amicon) employing urea as a low-mol-wt. marker.

*Target cells.*—Four pairs of cells were examined. They were normal and malignant cells from 4 species chosen to provide matched pairs with similar doubling times in conventional medium (containing 200 μg/ml L-arginine). All the cells were tested at low passage number and were constantly replenished from low-passage stocks maintained in liquid $N_2$. The cells employed were:

(a) *Human*: HYP66T, derived from a surgical specimen of hypernephroma, which has characteristic epithelioid morphology with lipid cytoplasmic granules, shows no evidence of contact inhibition and produces tumours in immune-deprived mice (A. Strain, personal communication). NK66 is a cell derived from the normal renal cortex of the uninvolved kidney from the same patient, at the opposite pole from the tumour. These cells are uniformly epithelioid in monolayer culture.

(b) *Rat*: HSN is a benzpyrene-induced fibrosarcoma. The cultured cells are tumorigenic in syngeneic (hooded) rats. XIPH1 is a characteristic mesenchymal cell obtained by trypsinization of the xiphisternum of a normal August rat. It shows clear density-dependent inhibition of growth.

(c) *Mouse*: FS29, derived from a methylcholanthrene-induced C57BL fibrosarcoma, are characteristic sarcoma cells, show no contact inhibition and give rise to malignant tumours in the syngeneic host.

CBA (NK) cells were derived from a normal CBA mouse kidney and used as the normal control for the FS29 since it has a similar doubling time and shows density-dependent inhibition of growth.

(d) *Hamster*: baby-hamster-kidney cell lines transformed by polyoma virus (PYY) and untransformed (A3) (kindly provided by Dr S. Revell) show similar doubling times and plating efficiencies. However, the PYY cells show typical transformed colonial morphology and no contact inhibition, whereas the A3 cells have phenotypically normal features in monolayer cultures.

All the cells were maintained in RPMI 1640 containing 200 μg/ml L-arginine with 10% undialysed foetal bovine serum. They were grown in 25 $cm^2$ disposable plastic flasks, and were passaged when confluent with 0·1% trypsin and fed thrice weekly.

$^{125}$*IUdR incorporation*.—As a measure of DNA synthesis, the ability of cells to incorporate $^{125}$IUdR was estimated at varying concentrations of L-arginine. Cells obtained from stock cultures by trypsinization were inoculated in arginine-free RPMl 1640 plus 10% dialysed foetal bovine serum into the wells of 3040 Microtest II plates at $5 \times 10^3$ cells/well in 0·1 ml medium plus the appropriate concentration of arginine. After 68 h incubation at 37°C in a humid atmosphere containing 5% $CO_2$ the medium was gently aspirated and replaced with 100 μl of the appropriate fresh medium containing 37k Bq/ml$^{125}$ I-labelled IUdR (Radiochemical Centre, Amersham). The control cells (both normal and malignant) were in the log phase of growth when pulsed so that the effects of density- or contact-dependent inhibition would be minimized. The plates were then re-incubated for a further 4 h, after which the attached cells were washed × 3 and 0·2 ml of 1% alkaline sarkosyl NL97 (Geigy) added to each well. After 10 min at room temperature, 0·1 ml of the lysate was sampled and assayed for $^{125}$I activity in an automatic gamma counter.

*Cell numbers*.—Target cells were added in the appropriate medium to 25 $cm^2$ disposable culture flasks at $1{\cdot}5 \times 10^4$/ml in 10 ml medium. At appropriate times the total cell content of each culture was assessed by gentle trypsinization with 2 ml 0·1% crystalline trypsin (Armour) and the cell concentration counted in a haemocytometer under phase-contrast microscopy. The culture supernatant removed before trypsinization was centrifuged, and any unattached cells added to the trypsinized suspension before counting.

*Macrophages and supernatants*.—CBA female mice aged 10 weeks were injected i.p. with 1 ml thioglycollate medium, and peritoneal exudate cells collected 3 days later. These cells were added to 25 $cm^2$ disposable plastic flasks at $\sim 10^7$ macrophages per bottle (estimated from a prior count of adherent spreading cells) in RPMI 1640 containing 20 μg/ml L-arginine and 10% dialysed foetal bovine serum. Non-adherent cells were removed by vigorous washing after incubation for 1 h at 37°C. *E. Coli* lipopolysaccharide (Difco) was added at 25 μg/ml and the cultures incubated for 24 h at 37°C. The supernatant medium was then decanted and filtered through a 0·22 μm millipore filter before testing. Control supernatants were obtained by adding LPS to medium with no cells and incubating under the same conditions, or the incubation of macrophages with no endotoxin. Arginase content of macrophages and/or supernatants was assayed by the method of Herzfeld & Raper (1976). This is a colorimetric assay which involves the measurement of urea produced from L-arginine by the action of arginase, following activation with manganese ions.

### RESULTS

### *Effect of activated macrophage supernatants on $^{125}IUdR$ incorporation by target cells*

Supernatant media obtained from LPS-activated mouse macrophage monolayers contained arginase activity, as previously reported (Currie, 1978). These supernatants were then added in serial dilution to microplate cultures of 4 target cells. The final arginine concentration in the medium was 110 μg/ml. After 3 days' incubation, $^{125}$IUdR incorporation studies showed that the activated macrophage supernatants produced a dose-dependent inhibition of $^{125}$IUdR uptake by malignant cells but not by the normal cells (Fig. 1). The control media contained no detectable arginase activity and had no effect on $^{125}$IUdR incorporation. It is unlikely that extracellular factors such as those described by Opitz *et al.* (1975) can be incriminated in our results, since the active supernatants were removed before pulsing with $^{125}$IUdR and the cultures showed clear changes in cell numbers. As Fig. 1 shows, both PYY and HYP66T cells show substantial inhibition in $^{125}$IUdR uptake whereas the 2 normal cells examined, Xiph 1 and CBA(NK), were unaffected. Furthermore, the inhibitory activity of macrophage supernatant tested at 1:2 was completely abrogated by the addition of 2 mg/ml L-arginine.

### *Effect of L-arginine concentration on $^{125}IUdR$ incorporation*

Cultures were established in microplates in serial dilutions of L-arginine ranging from 200 down to 0 μg/ml and $^{125}$IUdR incorporation assayed 3 days later. Fig. 2 shows that the malignant cells require a higher concentration of L-arginine in the medium to maintain $^{125}$IUdR incorporation than do the corresponding normal cells; for example, FS29 cells required 100 μg/ml to maintain the level of incorporation shown by the "normal" CBA(NK) cells with 3 μg/ml of L-arginine.

FIG. 1.—Inhibition of $^{125}$IUdR uptake by cells exposed to supernatant from LPS-activated CBA mouse macrophages. Significant inhibition was obtained on neoplastic cells HYP66T ●——● and PYY ○——○) and not on "normal cells" (Xiph 1 ○– – –○ and CBA (NK) ●– – –●). Inhibition of $^{125}$IUdR uptake by neoplastic cells in 50% supernatant was completely abrogated by adding extra L-arginine (2 mg/ml).

### *Effect of complete arginine deprivation on cell numbers*

The mouse and hamster pairs of cells were cultured in medium containing either 200 μg/ml L-arginine or no arginine, and cell numbers were estimated daily. As the diagram (Fig. 3) shows, when cultured in arginine-containing medium both pairs of cells proliferated normally with similar doubling times. Without arginine, however, no cell proliferation occurred in any of the tested cells and the malignant or transformed cells from each pair (FS29 and PYY cells) both showed a substantially greater reduction in cell numbers, indicating that they die more rapidly than their normal counterparts in an arginine-free environment.

FIG. 2.—Effect of L-arginine concentration on $^{125}$IUdR uptake by normal and neoplastic cells. FS29 ○——○, CBA(NK) ●- - -●, HSN □——□, Xiph 1 ■- - -■.

FIG. 3.—Effect of culturing mouse and hamster cell pairs in arginine-free medium for 3 days, showing poorer survival of the neoplastic cells. Mouse cells: FS29 with arginine ○——○, without arginine ○- - -○, CBA(NK) with arginine ●——●, without arginine ●- - -●. Hamster cells. PYY with arginine ○——○, without argine ○- - -○, A3 with arginine ●——●, without arginine ●- - -●.

*Time course of rescue of arginine-deprived cells*

All 4 pairs of cells were cultured in arginine-free medium and at Time 0 and at intervals thereafter the cells were rescued by the addition of 200 µg/ml L-arginine. The cells were then cultured for 4 days (following rescue) and the number of cells in the culture counted. This is an assay of the effect of timed periods of arginine starvation on subsequent reproductive capacity of the cells. As Fig. 4 indicates, there were marked differences between the normal and malignant cells in each pair. The malignant cells rapidly lost their proliferative capacity and could not be rescued by the addition of L-arginine after 24–36 h, whereas the normal cells withstood arginine deprivation for longer and growth inhibition was reversible.

### DISCUSSION

Most mammalian cells will not proliferate in tissue culture without a source of exogenous L-arginine (Eagle, 1959). Arginine deprivation kills cells, and the studies reported above clearly reveal a quantitative difference between the normal and malignant cells tested in their requirements for exogenous arginine. Since the cytotoxic action of activated macrophages and of their supernatant media can be attributed to arginase-mediated arginine depletion (Currie, 1978), the selectivity of this cytotoxicity (Currie & Basham, 1975) can in our hands be explained solely on the basis of differences in arginine requirements. While there may be other mechanisms whereby activated macrophages influence the proliferation and survival of target cells, the temporal kinetics of target-cell destruction by activated macrophages (Alexander & Evans, 1971; Currie & Basham, 1975) are strikingly similar to those induced by the

FIG. 4.—Effect of increasing periods of complete arginine starvation on subsequent growth of cells after rescue with 200 μg/ml L-arginine, showing irreversible growth inhibition of the neoplastic cells from each pair. Neoplastic cells ○– – –○, Normal cells ●——●.

addition of bovine liver arginase or by culturing the cells in an arginine-free environment (unpublished observations). Under other test conditions the generation of complement breakdown products or the release of polyamine oxidase have been incriminated (Allison, 1978). The lysis of target cells induced, for instance, by exposure to C3a (Schorlemmer *et al.*, 1977) is extremely rapid and, as the authors stated, more closely resembles the effects of natural-killer (NK) cells than the "classical" activated macrophage.

The reasons for cell death due to arginine deprivation are obscure, but 2 major pathways deserve consideration:

(1) *Interference with polyamine biosynthesis.* Arginine, in the urea cycle, is the precursor for ornithine biosynthesis, and arginine depletion must lead to reduced intracellular levels of ornithine which in turn could curtail polyamine biosynthesis. Polyamines are essential triggers for nucleic-acid synthesis, and reduction in polyamine production by limiting ornithine supplies causes dramatic inhibition of RNA and DNA synthesis. However, the previously published study (Currie, 1978) showed that cytotoxicity due to arginine deprivation could not be abrogated by the addition of L-ornithine or even putrescine. Furthermore the action of extracellular arginase on L-arginine provides a source of ornithine for the polyamine pathway.

(2) *Interference with protein synthesis.* When citrulline replaced arginine in the culture medium, cell proliferation proceeded normally. We therefore conclude that the target cells can synthesize arginine from citrulline *via* arginino-succinate, and that restriction of arginine probably prevents the biosynthesis of proteins essential for cell survival. Deprivation of single amino acids including arginine induces severe chromosome abnormalities in cultured cells (Freed & Schatz, 1969). Growth inhibition by arginine depletion is associated with continued initiation of DNA synthesis (Weissfeld & Rouse, 1977) but severely depressed protein and RNA synthesis (Weinberg & Becker, 1970). This type of metabolic imbalance (although the other way round) is reminiscent of the effect of thymidine excess on cultured cells, which also causes severe chromosome damage (Yang *et al.*, 1966) and is selectively cytotoxic for malignant cells (Lee *et al.*, 1977).

The difference in arginine requirements of the normal and malignant cell pairs were revealed in 2 ways. Firstly, the malignant cells examined required higher levels of L-arginine to maintain optimal cell proliferation and DNA synthesis. Secondly, the capacity of cells to withstand increasing periods of complete arginine depletion also revealed a significant difference, in that the cytostatic effect of arginine deprivation on normal cells was more readily reversible than on their malignant counterparts. Cells such as the L5187Y lymphoma grow well in media containing low concentrations of L-arginine, as do V79 Chinese hamster lung cells, and yet both these cells are highly susceptible to the cytotoxic activity of macrophage supernatants or short periods of total arginine starvation (Currie, 1978). Such observations suggest that the basis for selective cytotoxicity may reside in the capacity of normal cells to withstand relative arginine deprivation better than malignant cells, rather than in differences in their absolute quantitative requirements for exogenous arginine.

Bach & Lasnitzki (1947) showed that slow-growing tumours contain more arginase than fast-growing tumours and they suggested that arginase represents some kind of natural defence against malignant cells. The arginase concentrations in tumours in general are said to be much higher (Roberts & Frankel, 1949) than parallel normal tissues (with the exception of liver and kidney which possess an intact urea cycle). Preliminary studies in this laboratory reveal that macrophages freshly isolated from a tumour contain high levels of arginase, and that malignant cells derived from the same tumour contain very low levels. We believe that the tumour levels of arginase, as reported by Bach & Lasnitzki (1947) may have been associated with host-macrophage infiltration. The presence of abundant free L-arginine *in vivo* would not rule out a local microenvironmental role for arginine depletion in a macrophage-rich tumour or a granuloma. An examination of this topic will be published separately.

Storr & Burton (1974) have previously described selective cytotoxic effects of arginine deprivation, in that murine lymphoma cells die rapidly in arginine-free medium whereas normal syngeneic thymocytes can survive for much longer. Lymphocyte transformation is highly susceptible to arginine deprivation, exposure to mycoplasma arginine deiminase or the addition of an arginine analogue (Simberkoff *et al.*, 1969) and a role for arginase release in the effects of suppressor macrophages is suggested by the work of Kung *et al.* (1977). Unlike malignant cells, however, the inhibition of lymphocyte blastogenesis by prolonged arginine deprivation is readily reversible (Storr & Burton, 1974).

Activated macrophages are selectively cytotoxic to malignant cells. We suggest that the biological basis for this selectivity may reside in the arginine-dependence of the target cells. Cytotoxic effects on virus-infected cells (Goldman & Hogg, 1977) and on microorganisms may have a similar basis.

These studies are supported by a programme grant from the Medical Research Council. G.A.C. thanks the Cancer Research Campaign for financial support.

### REFERENCES

ALEXANDER, P. & EVANS, R. (1971) Endotoxin and double-stranded RNA render macrophages cytotoxic. *Nature (New Biol.)*, **232**, 76.

ALLISON, A. C. (1978) Mechanisms by which activated macrophages inhibit lymphocyte responses. *Immunol. Rev.*, **40**, 3.

BACH, S. J. & LASNITZKI, I. (1947) Some aspects of the role of arginine and arginase in mouse carcinoma 63. *Enzymologia*, **12**, 198.

CURRIE, G. A. (1978) Activated macrophages kill tumour cells by releasing arginase. *Nature*, **273**, 758.

CURRIE, G. A. & BASHAM, C. (1975) Activated macrophages release a factor which lyses malignant cells but not normal cells. *J. Exp. Med.*, **142**, 1600.

EAGLE, H. (1959) Amino Acid Metabolism in Mammalian Cell Cultures. *Science*, **130**, 432.

FREED, J. J. & SCHATZ, S. A. (1969) Chromosome aberrations in cultured cells deprived of single essential aminoacids. *Exp. Cell Res.*, **55**, 3939.

GOLDMAN, R. & HOGG, N. (1977) Enhanced susceptibility of virus-infected cells to starch-induced peritoneal exudate cells. in *The Macrophage and Cancer*. Ed. K. Jones, W. McBride and A. Stuart. Edinburgh: p. 97.

HERZFELD, A. & RAPER, S. M. (1976) The heterogeneity of arginases in rat liver. *Biochem. J.*, **153**, 469.

HIBBS, J. B. (1973) Macrophage non-immunologic recognition: target cell factors related to contact inhibition. *Science*, **180**, 868.

KUNG, J. T., BROOKS, S. B., JAKWAY, J. P., LEONARD, L. L. & TALMAGE, D. W. (1977) Suppression of *in vitro* cytotoxic response by macrophages due to induced arginase. *J. Exp. Med.*, **146**, 665.

LEE, S. S., GIOVANELLA, B. C. & STEHLIN, J. S. (1977) Selective lethal effect of thymidine on human and mouse tumour cells. *J. Cell. Physiol.*, **92**, 401.

OPITZ, H. G., NIETHAMMER, D., LEMKE, H., FLAD, H. D. & HUGET, R. (1975) Inhibition of $^{3}$H-thymidine incorporation of lymphocytes by a factor from macrophages. *Cell. Immunol.*, **16**, 379.

PIESSENS, W. F., CHURCHILL, W. H. & DAVID, J. R. (1975) Macrophages activated *in vitro* with lymphocyte mediators kill neoplastic but not normal cells. *J. Immunol.*, **114**, 293.

ROBERTS, E. & FRANKEL, S. (1949) Arginase activity and nitrogen content in epidermal carcinogenesis in mice. *Cancer Res.*, **9**, 231.

SCHORLEMMER, H. U., HADDING, U., BITTER-SUERMANN, D. & ALLISON, A. C. (1977) The role of complement cleavage products in killing of tumour cells by macrophages. In *The Macrophage and Cancer*, Ed. K. James, W. McBride and A. Stuart. Edinburgh: p. 68.

SIMBERKOFF, M. S., THORBECKE, G. J. & THOMAS, L. (1969) Studies of PPLO infection v. inhibition of lymphocyte mitosis and antibody formation by mycoplasmal extracts. *J. Exp. Med.*, **129**, 1163.

STORR, J. M. & BURTON, A. F. (1974) The effects of arginine deficiency on lymphoma cells. *Br. J. Cancer*, **30**, 50.

WEINBERG, A. & BECKER, Y. (1970) Effect of arginine deprivation on macromolecular processes in Burkitt's lymphoblasts. *Exp. Cell Res.*, **60**, 470.

WEISSFELD, A. S. & ROUSE, H. (1977) Continued initiation of DNA synthesis in arginine-deprived Chinese hamster ovary cells. *J. Cell Biol.*, **73**, 200.

YANG, S. J., HAHN, G. M. & BAGSHAW, M. A. (1966) Chromosome abberations induced by thymidine. *Exp. Cell Res.*, **42**, 130.